# A New Method to Predict Damage to Composite Structures Using Convolutional Neural Networks

**DOI:** 10.3390/ma16227213

**Published:** 2023-11-17

**Authors:** Laurent Mezeix, Ainhoa Soldevila Rivas, Antonin Relandeau, Christophe Bouvet

**Affiliations:** 1 Faculty of Engineering, Burapha University, 169 Long-Hard Bangsaen Road, Chonburi 20131, Thailand; laurentm@eng.buu.ac.th; 2 INSA Toulouse, 135 Avenue de Rangueil, CEDEX 4, 31077 Toulouse, France; soldevil@insa-toulouse.fr (A.S.R.); relandea@insa-toulouse.fr (A.R.); 3 INSA/ISAE-SUPAERO/IMT Mines Albi/UPS, Institut Clément Ader (CNRS UMR 5312), Université de Toulouse, 10 av. E. Belin, CEDEX 4, 31055 Toulouse, France

**Keywords:** Convolutional Neural Network (CNN), carbon fiber-reinforced polymer, composite, impact, impact damage

## Abstract

To reduce the cost of developing composite aeronautical structures, manufacturers and university researchers are increasingly using “virtual testing” methods. Then, finite element methods (FEMs) are intensively used to calculate mechanical behavior and to predict the damage to fiber-reinforced polymer (FRP) composites under impact loading, which is a crucial design aspect for aeronautical composite structures. But these FEMs require a lot of knowledge and a significant number of IT resources to run. Therefore, artificial intelligence could be an interesting way of sizing composites in terms of impact damage tolerance. In this research, the authors propose a methodology and deep learning-based approach to predict impact damage to composites. The data are both collected from the literature and created using an impact simulation performed using an FEM. The data augmentation method is also proposed to increase the data number from 149 to 2725. Firstly, a CNN model is built and optimized, and secondly, an aggregation of two CNN architectures is proposed. The results show that the use of an aggregation of two CNNs provides better performance than a single CNN. Finally, the aggregated CNN model prediction demonstrates the potential for CNN models to accelerate composite design by showing a 0.15 mm precision for all the length measurements, an average delaminated surface error of 56 mm^2^, and an error rate of 7% for the prediction of the presence of delamination.

## 1. Introduction

Carbon fiber-reinforced polymers (CFRPs) are widely adopted by numerous industries due to their high modulus and strength, and low density, which allow for weight reduction. Moreover, CFRPs show excellent fatigue resistance, creep, and corrosion resistance properties [1]. Despite their advantages, CFRPs’ vulnerability to impact is a significant concern [2,3,4]. Maintenance tool drops or debris impact during service can cause damage within the composite structure’s laminate, while leaving only subtle surface indentations [5]. Internal damage typically includes delamination, matrix cracking, and fiber rupture [6,7]. Low-velocity impact can considerably reduce a CFRP’s residual strength, especially its compressive strength [8,9,10,11]. Even though aramid fibers present good impact performance, the weak interfacial adhesion between aramid fibers and the resin matrix is considered a significant limitation to its utilization [12].

**Figure 1:**
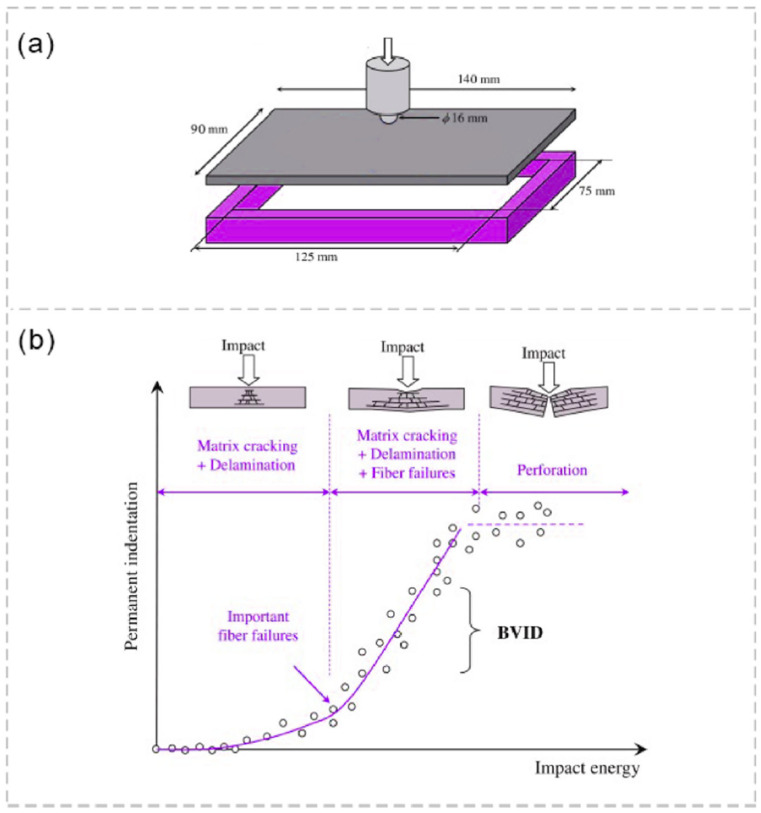
(**a**) Schematic of impact test setup, and (**b**) different impact damage stages with respect to the impact energy and permanent indentation size.

The issue of impact damage dictates a damage tolerance approach in the field of composite structure design to ensure that the structure is able to withstand in-service loads, even if the damage is undetectable. This design philosophy of impact damage tolerance has led to standards for composite coupons under low-velocity/low-energy impact [13] and Compression After Impact [5]. Figure 1 illustrates the impact geometry defined by the standard ASTM D7136 and various types of impact damage corresponding to different levels of impact energy [2]. In the initial stage, damage manifests as small matrix cracks as the impact causes minimal denting. As the impact energy increases, delamination occurs, leading to larger dents. In the second stage, all three types of damage—matrix cracks, delamination, and fiber breakage—can occur, making visual inspection easier due to the larger dent size (dent depth and diameter). The presence of fiber breakage in this stage aids in achieving more accurate inspection results. However, this can negatively impact the material’s residual strength after the impact, contributing to the complexity of the interaction between various damage types during an impact event. Finally, in the third stage, the damage becomes visible to the naked eye as perforation occurs.

The complex phenomena of composite damage, specifically those developing during impact loading, depend on several parameters, such as the matrix and fiber materials, the stacking sequence, the weaving pattern, the ply thickness, etc., and thus, make the use of models difficult [14,15] for the design of composite structures in the aeronautical field in terms of impact damage tolerance. In particular, it is necessary, at the same time, to simulate the damage developing during impact, the permanent indentation caused by the impact (which determines whether the damage can be detected during a visual inspection), and finally, the residual strength (in order to evaluate the loss of mechanical characteristics due to impact). Additionally, a lot of complex numerical models have been developed in the literature [4,16,17,18], but this requires a lot of knowledge and additional experiments in order to better study the proposed composite structures, and finally, there is a significant need for IT resources to run the models (mainly finite element models). Therefore, artificial intelligence could be an interesting way of sizing composites in impact damage tolerance studies. The initial phase involves simulating and predicting both the impact damage and the permanent indentation. These aspects are the focus of this article.

Machine learning, a subset of artificial intelligence, focuses on identifying patterns and correlations within large and diverse datasets. This approach involves a stochastic process and encompasses a wide range of algorithms, each striving to establish relationships in the data by performing various learning tasks. Among these algorithms, Artificial Neural Networks (ANN) are notable as universal approximators and are commonly employed for classification and regression tasks [19,20,21,22]. Another successful approach in image processing involves Convolutional Neural Networks (CNNs). Subsequently, CNNs have dominated the popular ImageNet challenge, achieving outstanding results across multiple evaluation metrics [23].

Among the various non-destructive testing (NDT) methods available for aerospace composite structures, visual inspection stands out as a widely used approach due to its rapid assessment of surface damage. Nonetheless, this method heavily relies on human-related factors, making its effectiveness susceptible to human judgment and subjectivity. Additionally, it may not be capable of detecting barely visible impact damage (BVID), which can be challenging to identify with the naked eye. Artificial intelligence (AI)-based techniques for detecting impact damage in composite materials can be broadly classified into three primary types: image-based, vibration-based, and acoustic-based methods. Image-based approaches employ computer vision algorithms to analyze images of the composite panel’s surface before and after an impact event, enabling the detection of surface topography changes, such as cracks and fiber breakage. Vibration-based methods focus on measuring and analyzing the composite structure’s vibrational response to identify changes in mechanical properties, including stiffness and damping, resulting from impact damage. Acoustic-based techniques utilize acoustic sensors to detect changes in acoustic emission signals generated by impact damage [22,24,25,26]. Numerous studies have investigated the application of AI-based methods for detecting impact-induced damage in polymer composite materials [27,28,29,30,31,32,33]. Beyond impact damage detection, AI-based techniques have wider applications in damage classification, damage quantification, and predicting the remaining useful life of composite materials [34]. These AI-driven approaches have the potential to advance impact damage assessment in composite materials, enhancing their reliability and durability. Tabatabaeian et al. successfully applied CNN models to detect BVID from both impacted and non-impacted surface images of composites [35]. In this study dataset, images were collected from impact tests carried out with energy from 3 J to 128 J, in a 32-ply CFRP composite [45/0/90/−45]_4s_ configuration. Both C-scan and visual inspection of the upper and lower surfaces were performed to create a dataset, and different CNN models were investigated. The predictions achieved an accuracy higher than 88.46% on the back face and a value between 51.25% and 97.05% for the impacted surface. In their study, Wei et al. [32] employed infrared thermography data from curved carbon fiber-reinforced polymer (CFRP) composites that had been impacted. They utilized these data to train two distinct deep learning models. These models successfully detected impact damage and accurately predicted the location of the damage, achieving an F1-score of 92.74% for mid-wave infrared data and 87.39% for long-wave infrared data. In their research, Hasebe et al. [30] utilized three machine learning models to analyze a dataset derived from low-velocity impact tests conducted on composites. Special attention was dedicated to three key factors: stacking sequence, impactor shape, and impact energy. The outcomes of their study revealed that characteristics such as local volume, dent surface gradient, and pure dent depth could effectively serve as indicators for characterizing internal damage in CFRP laminates.

The utilization of a machine learning-based approach holds immense potential in expediting the design process for optimal composite materials, resulting in significant time and resource savings [36]. The prediction of composite properties takes advantage of CNN models. The thermal conductivity properties of particle-filled 3D composites were predicted thanks to 2D CNN models using 3000 multiple cross-section images as the input [37]. The results showed that the use of five layers instead of three layers improved the accuracy of the CNN model. The elastic properties of composite materials, *E*_11_, *E*_22_ and *G*_12_, were predicted using a CNN model [38]. In order to create the dataset, the values corresponding to these properties were generated using an FEM. To address the significant computational resource challenge associated with generating training data, an innovative data augmentation scheme was introduced that enabled an increase in the dataset size from 9000 to 4.6 million samples. The results showed that the test error decreased from 2.4% to 0.4%. CNN models have been successfully used to predict the properties of composites beyond the elastic regime, i.e., crack propagation [39]. An FEM was used to obtain training data consisting of 26,000 configurations. Crack propagation under the quasi-static fracture of elastic solids was simulated using a hybrid formulation, and the elastic modulus, strength, and toughness were obtained from stress–strain curves. The results showed that CNN exhibited better performance than traditional models, i.e., linear regression and random forest. Stress–strain curves hold significance as they depict a material’s mechanical characteristics, outlining vital traits like the elastic modulus, strength, and toughness. The computational intensity escalates when generating these curves through numerical techniques like the finite element method (FEM), particularly when encompassing the complete failure trajectory of a material. The amalgamation of Principal Component Analysis (PCA) and Convolutional Neural Networks (CNN) has been employed to forecast the complete stress–strain curve of composite failures that extend beyond the elastic limit [40]. Using an FEM, a dataset containing 100,000 distinct composite microstructures and their corresponding stress–strain curves were created. This dataset was then utilized for both training and evaluating the performance of the model. The results showed a mean absolute error of less than 10%, demonstrating the robustness of the model. A comprehensive examination of an ANN in the modeling of composite materials was performed [41]. A large number of potentials used was identified: metamaterials [42,43,44], the mechanical behavior of yarns in textile composites [45], and the shape/size optimization of composite structures [46]. Finally, ANN models hold the potential to address a wide array of challenges, including unveiling unfamiliar physical principles and expediting computer simulations for composite materials.

In this work, CNN models to predict low-velocity/low-energy impact damage to FRP composites are explored. Initially, the data collection and preparation and the finite element procedure employed to acquire the training data for the CNN models are discussed. Then, two different CNN models are developed and optimized. Finally, the two CNN models’ results and validation are detailed and their performances are compared and discussed.

## 2. Methods

**Figure 2:**
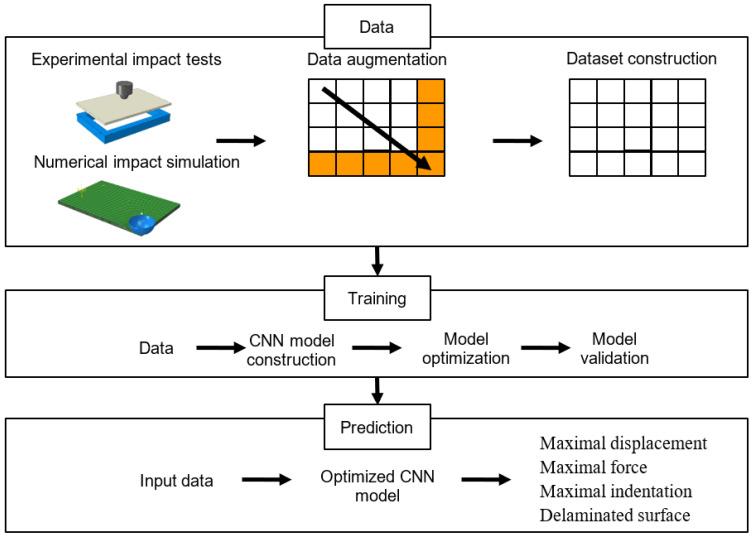
Overall methodology to predict impact damage to composites.

The approach to predicting impact damage to FRP composites used in this study can be summarized as follows (Figure 2):

Data: Data collection and creation, followed by data augmentation and dataset construction.

Training: Building, training, and validation of CNN models.

Prediction: Use of optimized CNN model to predict the impact damage to composites.

### 2.1. Data

The dataset was constructed by gathering information (data) identified as representing key factors that influenced the impact results of FRP [5,6,9,10]. The aim was to derive valuable insights into the mechanical behavior and performance of the various FRP materials subjected to impact. The data were organized into 3 types:

Sample parameters: Crucial details concerning the characteristics of the samples were captured, such as dimensions, stacking configurations, and material properties.

Impact test parameters: Vital information related to the impact test itself was compiled, particularly the impact energy and impact window size. This energy parameter played a crucial role in assessing the material response under dynamic loading conditions.

Impact test results: Impact tests results, encompassing pertinent metrics such as permanent indentation and maximum displacement, were collated.

To provide a comprehensive overview of the parameters used in the dataset, a list is provided in Table 1.

### 2.2. Dataset Construction

The dataset was built through spreadsheets where each influencing factor (Table 1) was arranged as a distinct feature in columns, while rows were employed to represent individual data source entries. To fully capture detailed information, ply characteristics were divided into four parameters: fiber orientation, material, thickness, and weaving type. As the largest composite lay-up consisted of 28 plies, a total of 112 columns were required to describe ply characteristics. Finally, the dataset consisted of 142 columns in order to cover all input information.

#### 2.2.1. Literature Data

The presented methodology involved a meticulous examination of the literature in order to collect impact tests results conducted on different materials. Content was based on over 11 different studies, with over 133 different impact tests conducted on the different materials [47,48,49,50,51,52,53,54,55,56]:

Fiber materials: carbon, Kevlar, and graphite fibers.

Resin materials: Epoxy, PEK, PPS, and PPS44.

Carbon fiber type: T700, T300, G30-500, ASA-8552, and IM7-8552.

Type of ply: prepreg or ‘dry’.

Core material: foam and honeycomb.

#### 2.2.2. Abaqus Model

**Figure 3:**
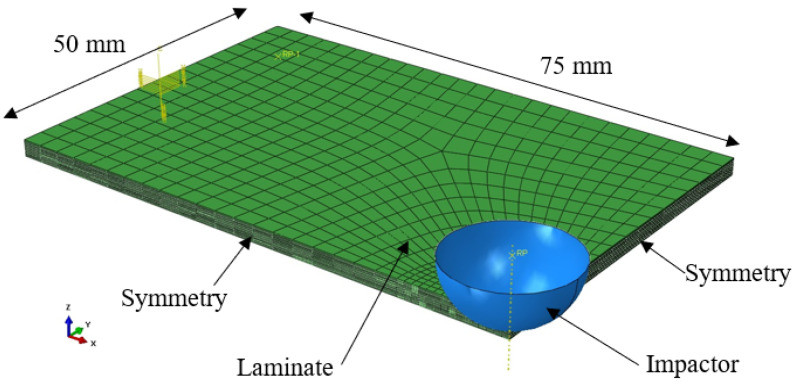
Geometry of the numerical model.

In order to enhance precision, additional data were needed; however, the available literature does not supply an adequate amount of data. To augment the dataset’s size, an ABAQUS model was employed. The final aim of the model was to be able to simulate the damage, and especially to obtain the delamination surface, maximum force, deflection, and indentation of laminates under low-velocity/low-energy impact. Therefore, the explicit model consisted of ply-by-ply laminate and the indentor (Figure 3). Normal behavior was used as a contact property in ABAQUS between the laminate and the indentor, while contact friction was neglected. As the indentor used was composed of a hardened steel, it was considered rigid [57]. Therefore, in the numerical model, the indentor was represented by an analytical rigid shell body. Due to the out-of-plane shear stress, solid elements were required; therefore, plies were modeled with C3D8 solid elements. In this study, 3 laminates were investigated: 8-, 12-, and 16-ply laminates of the same size (150 × 100 mm^2^). To reduce the computing time, only a quarter of the specimen was simulated (75 × 50 mm^2^) and symmetry conditions were imposed. A window of 125 × 75 mm^2^ was utilized on the lower surface of the laminate to fix the out-of-plane displacement.

In this first analysis, an FEA model was used to determine the impact energy required to reach the critical force avoiding delamination. Indeed, even though a lot of complex and relevant FEMs exist in the literature in order to simulate the damage that develops in composite structures during impact loading [1,2,3,4,5,6,7], the objective is to evaluate the ability of the AI to predict impact damage and its detectability. The critical force necessary to induce the beginning of delamination at the mid-thickness of the laminate under a mode II fracture is obtained using [6]:

(1) $$P_{c}^{2}=\frac{8\pi^{2}Eh^{3}}{9\left(1-\mu^{2}\right)}G_{IIc}$$

where *E* and *v* are the equivalent in-plane modulus and Poisson ratio for the laminate, and *h* is the laminate thickness. *G_IIc_* is the fracture toughness in mode II of the composite laminate and is taken to be equal to 1.5 N/mm [58]. This value depends on the composite material, but in this first approach, this value was kept constant in order to highlight the effect of the stacking sequence on the impact damage. As no damage was simulated, a simple elastic model was used in order to easily and quickly generate additional data to feed the AI, and linear elastic properties of unidirectional T700 carbon/epoxy ply were chosen (Table 2).

A large number of different stacking configurations were tested using Abaqus. The stacking of 8, 12, and 16 plies was investigated, and the stacking rules followed the aerospace design principles used in industry [60]. A total of 43 laminate stackings were investigated (Table 3). For each configuration, the FEA model enabled us to obtain the impact energy given the maximum force matching the one obtained using Equation (1).

#### 2.2.3. Data Augmentation

The performance of a CNN model relies on various factors, with the dataset being a crucial one [39]. Boosting the size of the dataset can greatly improve the prediction accuracy of the machine learning model [61]. However, generating an adequately large training dataset from an FEM can be computationally demanding and time-consuming, necessitating high computing resources. To address this challenge, a data augmentation strategy is presented to expand the dataset size substantially, thus reducing the computational resources needed during training. The data augmentation process is divided into two categories:

Symmetry: Experimental samples with a square or circle impact window show symmetry; therefore, rotation of 90° was applied as the properties remain the same as the original. This procedure increases the size of the base dataset of 24 lines.

**Figure 4:**
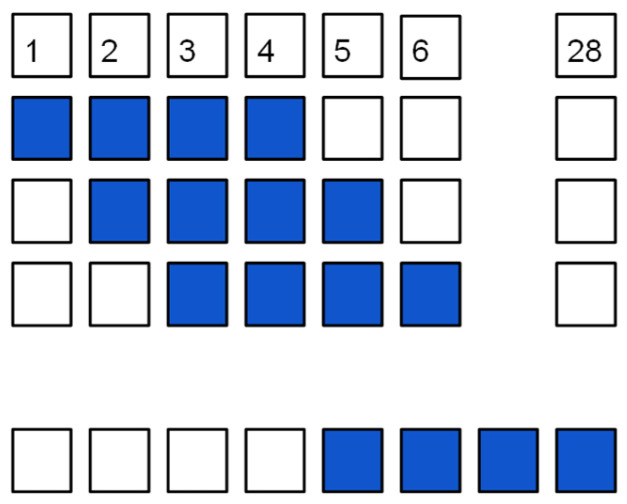
Translation of the layers.

Layer translation: The maximum number of composite layers is 28, but many of the investigated composites have only 12 or 16 layers. To ensure the model understands the usability of all layers, not just the initial ones, data entries of fewer than 28 layers were shifted to the subsequent layer, creating new data, as depicted in Figure 4. Using this method, the final total number of lines reached a value of 2725.

### 2.3. Input and Output Definitions

**Figure 5:**
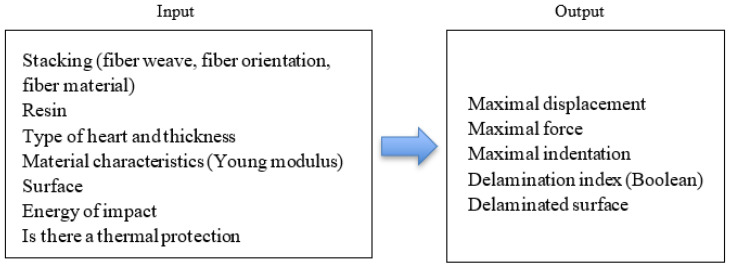
Inputs and outputs of CNN models.

In order to use the CNN models, data inputs and outputs were determined and were divided in the following way (Figure 5):

All textual inputs, such as fiber weaving pattern, material type, and state (prepreg or dry), underwent encoding using one-hot encoding. A similar approach was employed for non-continuous variables, such as fiber orientation. Since only certain values (e.g., 0 and 90) were valid orientations, one-hot encoding was applied to represent these admissible values. For numerical features, normalization was performed by dividing each feature by the maximum value within our dataset. This normalization procedure constrained the value range of each feature between 0 and 1. These preprocessing steps collectively ensured that each data point could be represented as a set of floating-point numbers, spanning an interval from 0 to 1. Subsequently, the data were structured according to a 2D grid to maximize the pattern detection of the CNN, where columns correspond to distinct layers, and rows denote the composite layer properties (Table 4). Scalar values were incorporated as rows within this matrix, repeating the same value 28 times to occupy the entire row uniformly. The dataset was configured as a float32 tensor, possessing dimensions of 2725 samples × 42 properties × 28 layers. The normalization process was extended to the outputs, ensuring the predictions aligned with the same value range.

In terms of dataset processing, comprehensive and reproducible shuffling of the tensors was executed. This meticulous shuffling guaranteed homogeneity during the CNN training process, while simultaneously preserving the association of inputs with their corresponding expected outcomes. Subsequently, the tensors were sliced based on a split percentage. By default, 90% of the data were allocated for training, and the remaining 10% were reserved for validation purposes.

### 2.4. CNN Model’s Construction

The networks were trained under a cloud computing environment using Python v3.10.12 and Tensorflow v2.12.0.

#### 2.4.1. Description of the First Supervised Network

A series of tests were run in order to select the best configurations for the network among more than 1700 different architectures. Specifically, 1, 2, 4, and 6 convolutional layers were evaluated, employing ascending or descending combinations of 4, 8, 16, 32, 64, 128, and 256 nodes. Independent kernel sizes ranging from 3 × 3 to 15 × 15 were also considered.

Optimization of the model parameters was achieved using the Adam optimizer, utilizing Keras’ default implementation. The loss function chosen was the mean absolute error, with its default Keras implementation chosen for its simplicity and for the low amount of outliers in the dataset. Among the hyperparameters influencing model training, the batch size holds significance. Integral to the optimization algorithm, it dictates the quantity of training samples processed before the internal model parameters receive updates. After trial and error, the default batch size of 32, as offered by the Keras library, yielded optimal outcomes. To forestall overfitting, the maximum epoch count was capped at 200, and a preventive strategy based on early stopping was implemented. Specifically, training terminated if the validation loss remained stagnant for 10 consecutive epochs. The ultimate weights chosen were those associated with the most favorable overall validation loss.

**Figure 6:**
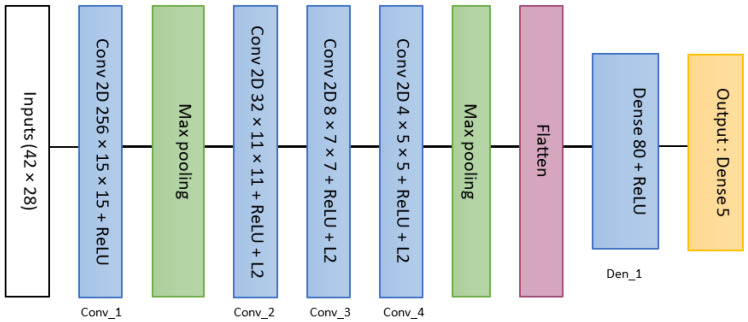
Flow chart of the first CNN architecture.

The best model necessitates 4 convolutional layers, comprising 256, 32, 8, and 16 nodes, followed by a fully connected layer housing 80 nodes (Figure 6). To mitigate overfitting risks, 2 max pooling operations were employed with a pooling rate of 0.5. Additionally, a rectified linear unit (ReLU) activation function was applied to each convolutional and fully connected layer. The convolutional layer kernels adhere to a descending logic, with dimensions of 15 × 15 for the initial layer, and subsequent sizes of 11 × 11, 7 × 7, and 5 × 5. All convolutional layers incorporate L2 regularization, employing a regularization parameter of 0.001, except for the first layer.

#### 2.4.2. Description of the Second Supervised Network (TwIN_Z6_Net)

**Figure 7:**
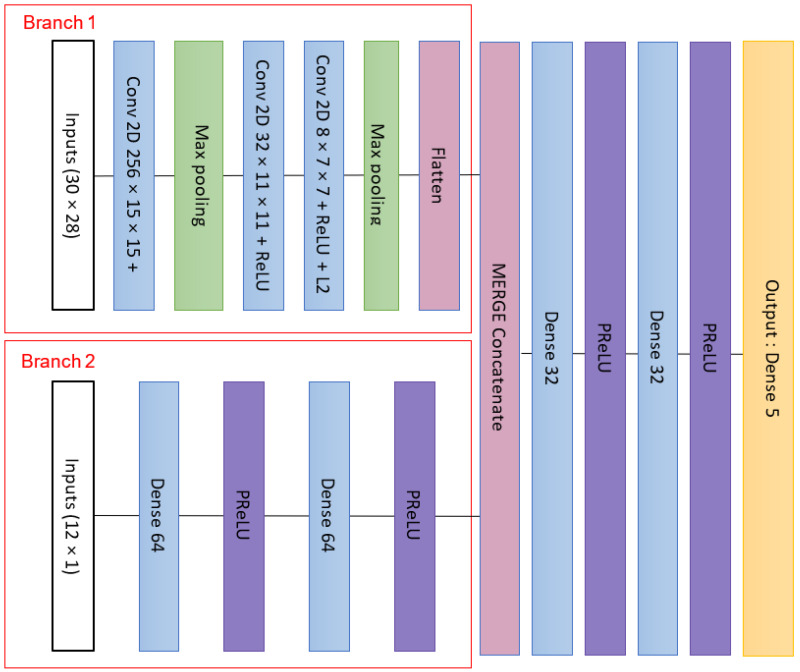
Flow chart of the second CNN architecture (TwIN_Z6_Net).

The input data were structured according to a 2D grid (Table 4). However, it can be observed that 2 types of data were used: single scalar representing data such as core presence, material composition, or impact energy values, and a matrix representing the composite stacking. However, in the context of stacking, deeming a scalar as a layer property appeared illogical. Therefore, in order to differentiate the types of inputs, a different approach was proposed. The inputs were separated into two parts: a matrix representing only the composite stacking, and a vector containing scalar parameters. These two parts were then processed through different paths in a Convolutional Neural Network (CNN). Afterward, the outputs from these different paths were combined and run through a simple Artificial Neural Network (Figure 7). This approach allowed us to generate the original outputs while addressing the challenge of incorporating diverse types of data. The initial branch (branch 1) is an extension of the previous model, where this pathway was streamlined by eliminating the final convolutional layer. This adjustment aimed to simplify the model and reduce training duration. Meanwhile, the second branch encompasses a basic structure consisting of a two-layer Artificial Neural Network (ANN) featuring 64 nodes in each layer, activated via the Parametric Rectified Linear Unit (PReLU) activation function. The fusion of these two branches occurs through concatenation, forming the input for two 32-node fully connected layers, also utilizing PReLU activation. PReLU was chosen as an enhancement over standard ReLU activation. This choice was driven by the aim to retain information embedded within the negative activation of neurons, all while preserving the nonlinear characteristics.

## 3. Results and Discussion

### 3.1. First Supervised Network

**Figure 8:**
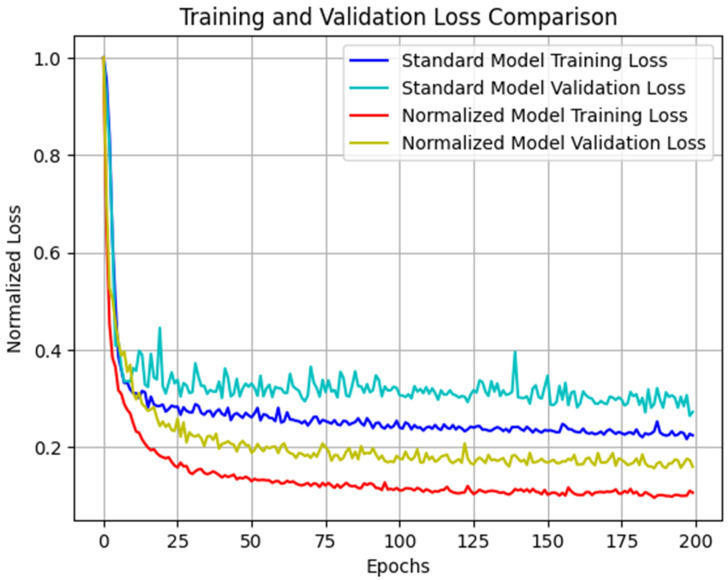
Training and validation normalized loss comparison.

Initially, two models based on the proposed CNN architecture were trained to conduct a comparative analysis of outcomes with and without the normalization of output data. The training process encompassed 90% of the dataset, with identical samples employed for training both models. The remaining subset was dedicated to validation purposes. Loss history was obtained, as shown in Figure 8, divided by the maximum loss value to allow for a comparison.

Upon completing the training process, the average error across all outputs using the validation data was computed. The analysis revealed that the normalized model exhibited superior overall performance (Table 5). Notably, the marginal reduction in precision for the delaminated surface output, while present, was deemed insignificant in relation to the actual value of the result. Indeed, the concept of scaling or normalizing outputs by dividing them by the maximum value of the dataset is often applied based on common sense or specific needs [62].

### 3.2. Second Supervised Network

**Figure 9:**
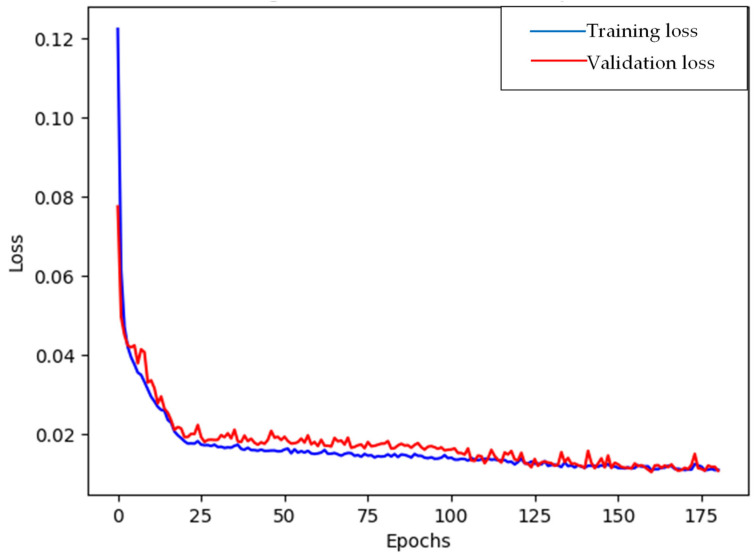
Training loss and validation loss per epoch.

Due to the previous result, output normalization was applied to the TwIN_Z6_Net model. Figure 9 illustrates the progression of the loss value throughout the training of the proposed architecture. The utilization of the Adam optimizer facilitates rapid convergence of the model towards the minimum loss value. Notably, the early-stopping mechanism was invoked around epoch 180 to avoid overfitting.

**Figure 10:**
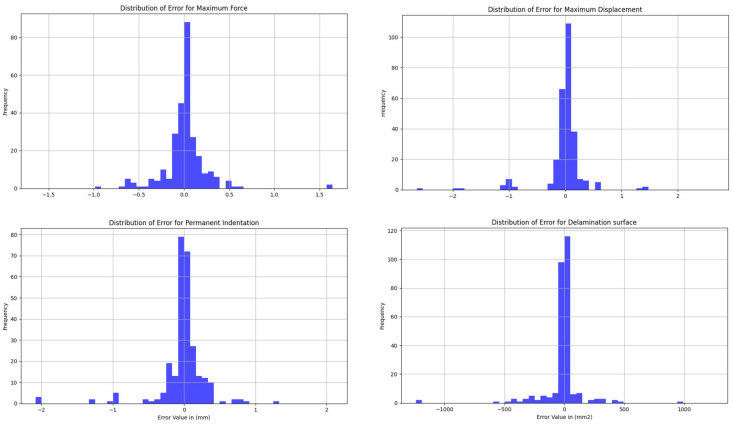
Distribution of the error across all the model outputs.

For the evaluation phase, the mean error of each output was calculated (Table 5). A clear improvement for all outputs can be observed. The highlight of our model is reaching 0.15 mm precision for all the length measurements. The error for each individual sample within the validation dataset was assessed, and we subsequently calculated both the mean and standard deviation for each output (Figure 10):

Output maximum force—mean: 0.0116 kN, std dev: 0.2451

Output maximum displacement—mean: −0.0132 mm, std dev: 0.3706

Output permanent indentation—mean: −0.0170 mm, std dev: 0.3458

Output delamination surface—mean: −14.4754 mm^2^, std dev: 164.2299

**Figure 11:**
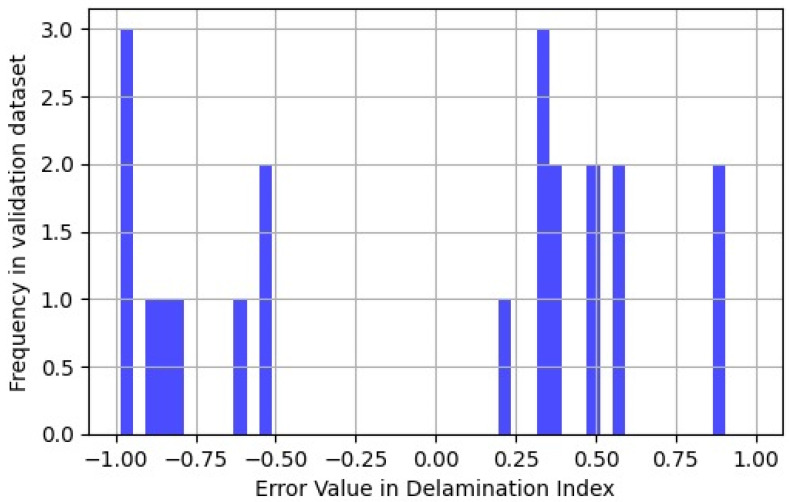
Distribution of errors for delamination index.

The treatment for the delamination index varied due to its binary nature, denoting the presence or absence of delamination on a sample. A slight post-processing step was introduced to enhance the interpretability of the floating-point output, enabling an easier assessment of its proximity to 0 or 1. This involved computing the absolute error value and applying a threshold filter, resulting in a Boolean outcome that reflects prediction accuracy. In this work, it was considered that there is no delamination for an index value lower than 0.2. It was observed that 11 samples without delamination were predicted to be delaminated, and conversely, 9 samples with actual delamination were predicted to have none (Figure 11). In total, the global error reached 20 out of 273 samples, yielding an error rate of 7.3%.

### 3.3. Perspective

**Figure 12:**
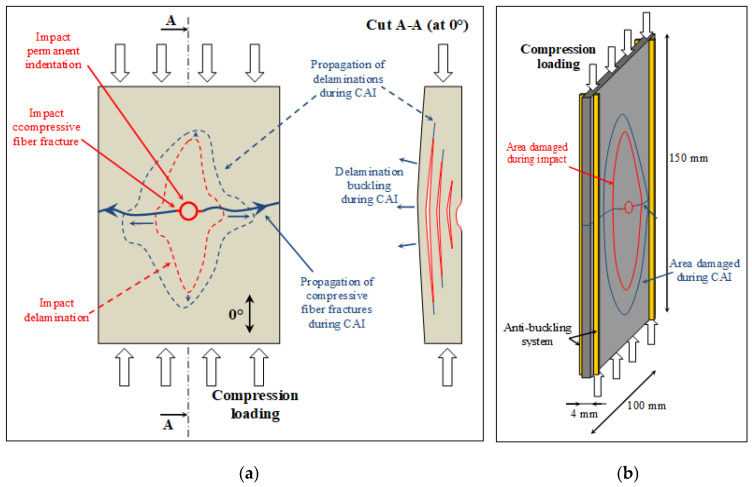
(**a**) Compression After Impact test set-up and (**b**) schematic damage that developed during Compression After Impact test.

The most intriguing aspect of designing a composite aeronautical structure for impact damage tolerance is not the impact itself, but rather, the loss of residual strength resulting from the impact, along with the detectability of the impact. Residual compressive strength after impact is a critical design factor in assessing the damage tolerance of fiber-reinforced polymers (FRP) for structural applications in aircraft, as it is classically the most affected mechanical characteristic by impact damage (due to the buckling of the delaminated plies) (Figure 12a). The Compression After Impact (CAI) test is a standardized method for characterizing the residual compressive strength for laminates of FRP, employing the ASTM D7136 [13] and ASTM D7137 [63] standards (Figure 12b). The full procedure follows two stages, where samples are first subjected to low-velocity impact via drop-weight impact testing and are, then, subjected to in-plane compressive loading using CAI apparatus. To design and optimize a composite structure for impact damage tolerance, it is essential to simultaneously simulate the damage development during impact, the permanent indentation left by the impact, and ultimately, the residual strength. While the numerical models presented in the literature [4,16] are highly valuable, their effective utilization demands extensive knowledge, additional experiments for a more in-depth examination of the composite structures under study, and a considerable number of IT resources to run the models. Applying the same methodology to an experimental/FEA dataset, proposing a CNN model for Compression After Impact (CAI) represents an intriguing approach for sizing composites in terms of impact damage tolerance. This method circumvents the need for FEA iteration, thereby reducing design calculation time. Designers would only be required to input parameters such as composite stacking definition and impact energy, and the CNN model would instantly provide the desired response.

## 4. Conclusions

Low-velocity/-energy impact on a composite structure has the characteristic of causing extensive internal damage within the layers of the material, despite only causing a slightly perceptible indentation on the surface. This type of damage results in a decrease in residual strength post-impact, particularly in terms of its compressive strength. Within the field of aeronautics, this reduction in strength compels designers to consider damage tolerance and restrict the utilization of the material’s full capabilities. As a result, accurately predicting the effects of impact damage has become crucial for enhancing the remaining strength of composites. In this work, CNN models are proposed to predict impact damage in FRP composites. On one hand, data are collected from the literature, while on the other hand, the finite element method (FEM) is employed to simulate impact, with an initial literature-to-FEM data ratio of 32%. A large amount of data information is gathered, and a data augmentation method is proposed to increase the data number from 149 to 2725. The data are divided into two parts: input and output data. The first one consists of a composite stacking definition and of the impact test parameters. The second one concerns the damage to the FRP composite, including maximum force, maximum displacement, indentation, delamination area, and delamination index. Then, two different CNN models are investigated and optimized. The first one is based on a traditional architecture and the second consists of an aggregation of two CNNs. The proposed method enables us to predict the impact damage to an FRP composite for given stacking configurations. However, to obtain the best results, the aggregation of two CNNs should be considered as it shows better performance than the traditional architecture—the first to process a matrix representing composite stacking and the second to process a vector containing scalar parameters (sample surface, impact energy, etc.). Moreover, normalization of the output data improves the CNN model’s performance. The best model reaches 0.15 mm precision for all the length measurements and an average delaminated surface error of 56.36 mm^2^, and a 7% error rate is obtained on the delamination index.

This work could facilitate the process in the area of damage tolerance design by providing rapid damage prediction for CFRP composite solutions. Furthermore, it has the potential to decrease the time and expenses associated with investigating and formulating novel FRP composites. In order to improve the model, work is in progress in order to increase the dataset size. Moreover, deeper analysis of the model regarding the influence of each parameter is in progress.

## Figures and Tables

**Table 1:** List of the dataset parameters.

Samples Parameters	Impact Test Parameters	Impact Test Results
In-plane Young’s modulus (GPa)	Impact window (mm^2^)	Permanent indentation (mm)
Stacking (orientation of each ply)	Energy of impact (J)	Maximum displacement (mm)
Laminate thickness (mm)	Symmetrical test plate? (1 for yes, 0 for no)	Maximum force (kN)
Material reference (T700...)	Is the test dynamic or static? (1 for dynamic, 0 for static)	Delaminated area (mm^2^)
Type of carbon (pre-impregnated or dry)		Is there perforation? (1 for yes, 0 for no)
Thermal protection (1 for yes, 0 for no)		Is there delamination? (1 for yes, 0 for no)
Resin type (epoxy…)		
Resin proportion (integer between 0 and 1)		
Resin property G_IIC_ (N/mm)		
Number of plies (integer)		
Sandwich core type (honeycomb, foam…)		
Sandwich core thickness (mm)		
Fiber weaving (woven or unidirectional)		

**Table 2:** Mechanical properties of unidirectional T700 carbon/epoxy [59].

	Properties
Tensile modulus (GPa)	135
Tensile strength (MPa)	2550
Compression strength (MPa)	1470
Flexural modulus (GPa)	120
Flexural strength (MPa)	1670

**Table 3:** Laminate stacking investigated through explicit FEA.

Number of Plies	Stacking	Energy of Impact (J)
8	[45/−45/45/−45]s	2.3
	[45/−45/45/−45]s	4
	[0/45/−45/90]s	4
	[0/45/−45/90]s	9
	[0/45/−45/90]s	11.5
	[0/90/0/90]s	11.5
	[0/90/0/90]s	16.8
	[45/90/−45/90]s	3.6
	[45/0/−45/0]s	13.6
	[45/0/−45/0]s	18.48
12	[45/−45/−45/45/0/0]s	8.992
	[45/−45/90/90/90/90]s	1.688
	[45/−45/−45/45/0/0]s	10.8
	[45/−45/−45/45/90/90]s	2.558
	[45/−45/0/0/0/0]s	15.9
	[45/−45/−90/−45/45/0]s	6.244
	[0/0/45/−45/90/90]s	10.23
	[0/45/−45/−45/45/90]s	6.244
	[0/0/0/45/−45/90]s	15.985
	[90/90/90/45/−45/0]s	5.755
	[0/0/45/−45/90/90]s	12.948
	[0/45/−45/−45/45/90]s	8.402
	[0/0/0/45/−45/90]s	18.473
16	[45/−45/0/−45/45/45/−45/90]s	2.248
	[45/−45/0/0/−45/45/90/90]s	2.25
	[45/0/−45/−45/0/45/92/90]s	2.247
	[−45/45/0/0/0/45/−45/90]s	2.247
	[45/0/0/−45/−45/90/90/45]s	2.247
	[−45/0/0/45/90/90/90/45]s	2.249
	[45/0/0/0/0/−45/90/90]s	7.84
	[45/0/−45/90/−45/0/45/90]s	7.83
	[45/−45/0/−45/45/45/−45/90]s	3.237
	[−45/0/0/45/90/90/90/45]s	6.243
	[45/−45/0/−45/45/45/−45/90]s	3.996
	[45/−45/0/0/−45/45/90/90]s	7.83
	[45/0/−45/−45/0/45/90/90]s	7.831
	[−45/45/0/0/0/45/−45/90]s	6.242
	[45/0/0/−45/−45/90/90/45]s	6.243
	[45/−45/0/−45/45/45/−45/90]s	4.4
	[−45/45/0/0/0/45/−45/90]s	7.83
	[45/0/0/−45/−45/90/90/45]s	7.28
	[45/0/0/0/0/−45/90/90]s	8.989
	[45/0/0/0/0/−45/90/90]s	11.546

**Table 4:** Description of 2D grid for 8 plies [0/45/−45/90]s.

	Composite Layer							
	1	2	3	4	5	6	7	8
0° orientation	1	0	0	0	0	0	0	1
+45° orientation	0	1	0	0	0	0	1	0
−45° orientation	0	0	1	0	0	1	0	0
90° orientation	0	0	0	1	1	0	0	0
Unidirectional or woven	1	1	1	0	0	1	1	1
Carbon fibers	1	1	1	1	1	1	1	1
Glass fibers	0	0	0	0	0	0	0	0
Kevlar fibers	0	0	0	0	0	0	0	0
Graphite fibers	0	0	0	0	0	0	0	0
Prepreg or ‘dry’	1	1	1	1	1	1	1	1
Core material	0	0	0	0	0	0	0	0

**Table 5:** Comparison of the different CNNs’ average errors.

	Average Error of Maximum Force (kN)	Average Error of Maximum Displacement (mm)	Average Error of Permanent Indentation (mm)	Average Error of Delamination Index	Average Error of Delaminated Surface (mm^2^)
Standard output	0.30	0.35	0.48	0.30	62.17
Normalized outputs	0.23	0.25	0.28	0.05	76.56
Improvement between standard and normalized output	0.07	0.1	0.2	0.25	−14.39
TwIN_Z6_Net	0.16	0.15	0.13	0.02	56.36

## Data Availability

The raw/processed data required to reproduce these findings are available upon request.
